# C-853-07. Under Pressure: Burn Specific Antibiogram, Ecology, and Antibiotic Use Compared to Surgical-trauma and the Institution

**DOI:** 10.1093/jbcr/irag033.075

**Published:** 2026-04-06

**Authors:** Scott W Mueller, Karrine Brade, Thomas O Vogler, Cameron Gibson, Alexandra Halevi, Fred Endorf, Arek J Wiktor

**Affiliations:** Burn & Frostbite Center at UCHealth, University of Colorado Hospital, Aurora, CO; UCHealth, Aurora, CO; University of Colorado, Aurora, CO; University of Colorado, Aurora, CO; University of Colorado School of Medicine, Aurora, CO; University of Colorado, Aurora, CO; Burn & Frostbite Center at UCHealth, University of Colorado Hospital, Aurora, CO

## Abstract

**Introduction:**

Burn sepsis guidelines suggest a unit specific antibiogram for empiric antibiotic choices. Despite clinical observations regarding the ecology of burn infections, additional data are needed to justify unit specific antibiograms. Our objective was to create a burn (BICU) specific Gram-negative (GN) antibiogram, compare the relative frequency of pathogens to a surgical trauma ICU (STICU) and the institutional antibiogram. We further assessed extended spectrum beta-lactam (Ex-BL) use mix in BICU vs STICU and the institution overall.

**Methods:**

Unit specific antibiograms and monthly antibiotic use patterns were collected at an ABA verified burn center over a 21 month period for BICU, STICU, and the institution overall. Escherichia spp, Klebsiella spp, Enterobacter spp, and *P. aeruginosa* counts were divided by overall GN counts to calculate rates of occurrence. Piperacillin-tazobactam (TZP), cefepime (FEP), and meropenem (MEM) are the primary Ex-BLs used with TZP being institutionally preferred. FEP and MEM were situationally restricted. Ex-BL mix was calculated by monthly antibiotic days / 1000 patient days with TZP as reference to normalize an intent to cover pathogens of interest. Difference in bacteria rate was analyzed by chi-squared and Wilcoxon-rank sum used to assess Ex-BL mix rates between BICU and STICU or institution.

**Results:**

GN bacteria differed between BICU, STICU, and the institution, respectively: Enterobacter spp 28%, 27%, and 10%; *P. aeruginosa* 20%, 18%, and 12%; Klebsiella spp 19%, 26%, and 20% and Escherichia spp 16%, 29%, and 52%; *p*<.01. This was driven by the difference between BICU and the institution (*p*<.01) as opposed to STICU, *p*=.08. Median antibiotic use was different between BICU and STICU for TZP (118 vs 200, *p*<.01), but not FEP (58 vs 37, *p*=.2) or MEM (40 vs 50, *p*=.07). FEP:TZP mix was different for BICU (45%) vs STICU (19%), *p*<.01, not the institution (43%). MEM:TZP mix was not different between BICU, STICU, or institution; 27%, 25%, 30%; respectively, *p*=.5. The overall probability of empiric susceptibility given a GN result for BICU (FEP = 87%, TZP = 84%, MEM = 94%) closely resembled the overall institution (FEP = 84%, TZP = 88%, MEM = 96%) than STICU (FEP = 75%, TZP = 77%, MEM = 96%) due to resistance patterns despite differences in bacteria mix between BICU vs institution and similarities between BICU vs STICU.

**Conclusions:**

BICU specific antibiogram confirmed striking differences in GN bacteria mix vs the institution. Yet empiric probabilities for empiric Ex-BL susceptibility in BICU resembled the institution, not STICU. The causal relationship between Ex-BL mix use and resistance requires further study.

**Applicability of Research to Practice:**

A BICU specific antibiogram revealed the inadequacy of an institutional antibiogram. It is a useful tool that may improve empiric antibiotic choices and allow for surveillance of trends over time.

**Funding for the study:**

N/A.

